# Genetic Modulation of *ATF1* in *Saccharomyces cerevisiae* for Enhanced Acetate Ester Production and Flavor Profile in a Sour Meat Model System

**DOI:** 10.3390/foods15020378

**Published:** 2026-01-21

**Authors:** Ning Zhao, Ying Yue, Shufeng Yin, Hao Liu, Xiaohan Jia, Ning Wang, Chaofan Ji, Yiwei Dai, Liguo Yin, Huipeng Liang, Xinping Lin

**Affiliations:** 1 State Key Laboratory of Marine Food Processing & Safety Control, National Engineering Research Center of Seafood, School of Food Science and Technology, Dalian Polytechnic University, Dalian 116034, China; ningzhao0221@163.com (N.Z.); nicoleyue1996@163.com (Y.Y.); yinshufeng1220@163.com (S.Y.); 18763666325@163.com (H.L.); jiaxiaohan1129@163.com (X.J.); ningwang0221@163.com (N.W.); jichaofan@outlook.com (C.J.); daiyiwei@dlpu.edu.cn (Y.D.); lhpdxx@126.com (H.L.); 2 Solid-State Fermentation Resource Utilization Key Laboratory of Sichuan Province, Yibin University, No. 5, Section 3 of Daxue Road, Sanjiang New Area, Yibin 644000, China; lgying22@126.com

**Keywords:** flavor formation, *Saccharomyces cerevisiae*, *ATF1*, acetate esters, fermented meat, transcriptomics

## Abstract

Acetate esters, synthesized by alcohol acyltransferase (AATases) encoded primarily by the *ATF1* gene, are pivotal for the desirable fruity aroma in fermented foods. However, the role and regulatory impact of *ATF1* in solid-state fermented meat remain largely unexplored. This study engineered *Saccharomyces cerevisiae* by knocking out and overexpressing *ATF1* to investigate its influence on flavor formation in a sour meat model system. Compared to the wild-type strain, *ATF1* overexpression (SCpA group) increased ethyl acetate content by 70.15% and uniquely produced significant levels of isoamyl acetate. Conversely, *ATF1* deletion (SCdA group) led to a 61.23% reduction in ethyl acetate. Transcriptomic analysis revealed that *ATF1* overexpression triggered a systemic metabolic shift, not only activating the final esterification step but also upregulating key genes in central carbon metabolism (*SUC2*, *ICL1*), amino acid biosynthesis, and precursor supply pathways (*ACS2*, *ADH1*). This synergistic regulation redirected metabolic flux towards the accumulation of both alcohol and acyl-CoA precursors, thereby amplifying acetate ester synthesis. Our findings demonstrate that *ATF1* is a critical engineering target for flavor enhancement in fermented meats and uncover a broader metabolic network it influences, providing a robust strategy for the targeted modulation of food flavor profiles.

## 1. Introduction

Fermented meat products are processed through microbial fermentation and biochemical actions. This not only imparts a unique flavor but also effectively extends the shelf life while improving the nutritional profile throughout the fermenting procedure. [1]. Sour meat, a traditional ethnic fermented meat product in China, is produced through a unique fermentation process. It is primarily made from pork, rice flour, and salt, and undergoes fermentation under anaerobic conditions. The final product is characterized by a firm texture and a distinct sour flavor with ester-like aroma notes. However, the characteristics of sour meat are influenced by a multitude of factors spanning ambient conditions, manufacturing procedures, duration, and material composition, which can lead to significant instability in quality [2]. The inoculation of starter cultures is an effective approach to enhance the stability and flavor profile of sour meat. For instance, Shang [3] inoculated *Lactiplantibacillus plantarum* and *Pediococcus pentosaceus* into fermented meat, significantly reducing the content of biogenic amines, enhancing its safety, and simultaneously stimulating the generation of volatile organic compounds. Guo [4] inoculated *Yarrowia lipolytica* into fermented meat, stimulating the synthesis of esters, aldehydes, and alcohols—with a notable increase in ethyl esters—thereby imparting a richer flavor profile to the product. Zhang [5] employed co-fermentation with *Latilactobacillus curvatus* and *Pediococcus pentosaceus* in fermented meat, which improved the color, total lactobacilli count, and free amino acid content, while the concentrations of nitrite, biogenic amines, total volatile base nitrogen (TVB-N), and malondialdehyde were effectively lowered. This led to a significant elevation in the safety and quality attributes of the fermented meat.

The ester content in fermented meat plays a dominant role in shaping its flavor profile. Crucial contributors include acetate and ethyl esters, with examples being ethyl acetate, ethyl octanoate, and ethyl hexanoate. These ester compounds typically contribute fruity and creamy flavors to fermented meat. Research has indicated that inoculating *S. cerevisiae* LXPSC1 in sour meat significantly improved the flavor quality, identifying it as an ideal fermentation agent for the enhancement of sour meat quality. *S. cerevisiae* shows a direct association with elevated concentrations of esters, alcohols, and total VOCs, with ester content in particular increasing by 4.36-fold. [6]. Research shows that S. cerevisiae is instrumental in ester compound synthesis due to the presence of AATases. AATases mediate the conversion of ethanol and acetyl-CoA into acetate ester. In *S. cerevisiae*, these AATases are primarily encoded by the *ATF1* and *ATF2* genes [7]. The synthesis of AATases is promoted by the overexpression of both the *ATF1* and *ATF2* genes [8,9]. This enhancement efficiently enables the condensation of acetyl-CoA with ethanol, yielding ethyl acetate as well as isoamyl acetate. Marullo assessed the roles of the *ATF1* and *ATF2* genes in regulating the synthesis of ethyl acetate and isoamyl acetate. The results indicated that *ATF1* plays a more critical role and is primarily responsible for producing both esters. In contrast, the absence of *ATF2* did not significantly affect the yield of either compound [10].

Traditional studies on microorganisms frequently employ intentional inoculation with specific microbial strains to investigate their impact on the attributes of fermented foods. However, the approach of adding microbial variants with gene knockouts or overexpressed genes is more conducive to thoroughly studying and clarifying the role of key genes in shaping the microenvironment. For instance, Shi [11] enhanced the production of the key enzymes acetyl-CoA synthase (*ACS1/2*) and aldehyde dehydrogenase (*ALD6*) in the Ac-CoA biosynthesis module by employing various terminators. Additionally, they achieved co-expression of the *ATF1* gene within the *ACS1/2* and *ALD6* cassettes, which led to a further increase in the ethyl acetate content of the liquor. Zhang [12] achieved a nine-fold increase in ethyl acetate production by overexpressing the *ATF1* gene through the transformation of a multicopy plasmid in industrial brewer’s yeast. The study by Dong [13] revealed that overexpression of the *ATF1* gene significantly boosts the strain’s capacity to produce ethyl acetate, thereby enriching the aroma of the liquid fermentation of corn hydrolysate and further improving its taste and flavor. The regulation of acetate esters by *ATF1* has been validated in liquid-state fermented foods, such as wine and beverages, particularly in alcoholic drinks. However, research on solid-state fermented foods, such as fermented meats, is exceedingly scarce.

Therefore, based on our preliminary findings regarding the impact of added *S. cerevisiae* on ester production in sour meat, we further investigated the influence of *ATF1*, a gene closely associated with ester synthesis in *S. cerevisiae*, on its flavor. We hypothesize that modulation of the expression level of the *ATF1* gene in *Saccharomyces cerevisiae* can significantly alter the synthesis efficiency of acetate esters, thereby directionally modulating the flavor profile of sour meat in a solid-state sour meat model system. In this study, we performed both knockout and overexpression of the *ATF1* gene, which encodes AATases in *S. cerevisiae*. The aim was to modulate the expression of ester synthesis-related genes at the genetic level in *S. cerevisiae*, and to examine the function of the *ATF1* gene in ester production in solid-state fermented foods. Furthermore, we utilized transcriptomic analysis to assess the effects of *ATF1* gene knockout and overexpression on the expression levels of other genes in *S. cerevisiae*. This aimed to uncover potential pathways and genes involved in ester synthesis, providing precise target information for further optimization of fermentation processes.

## 2. Materials and Methods

### 2.1. Experimental Materials, Strains and Plasmids

The pork belly and lard were obtained from the Dashui Market in Dalian City. Table 1 lists all the strains and plasmids used in this study.

### 2.2. Chemicals

All chemicals used were of analytical or specified grade. YPD liquid medium, agar powder, and LB liquid medium were obtained from Qingdao Hope Bio-Technology Co., Ltd. (Qingdao, China). Acetyl-CoA, amino acid mixture standard, and the following analytical standard esters: isoamyl acetate (CAS: 123-92-2), ethyl hexanoate (CAS: 123-66-0), ethyl octanoate (CAS: 106-32-1), ethyl butyrate (CAS: 105-54-4), ethyl acetate (CAS: 141-78-6), ethyl heptanoate (CAS: 106-30-9), ethyl decanoate (CAS: 110-38-3), phenethyl acetate (CAS: 103-45-7), and ethyl propionate (CAS: 105-37-3), as well as chromatographic grade cyclohexanone and C7–C30 saturated alkanes, were obtained from Sigma-Aldrich (St. Louis, MO, USA). Anhydrous ethanol and isoamyl alcohol (both HPLC grade) were supplied by Kemio Chemical Reagent Co., Ltd. (Tianjin, China). Trichloroacetic acid and acetone (chromatographic grade) were purchased from Aladdin Reagent (Shanghai) Co., Ltd. (Shanghai, China). The malondialdehyde (MDA) assay kit was provided by Nanjing Jiancheng Bioengineering Institute (Nanjing, China).

### 2.3. Knockout and Overexpression of the ATF1 Gene

The primers used for knockout and overexpression of the *ATF1* gene in *S. cerevisiae* SC are listed in Table S1. The *ATF1* knockout plasmid was constructed by amplifying fragments using primer pairs Ag-1 & Pg-1, Ag-2 & Pg-2, and P-6005 & P-6005. Subsequently, the three amplified products were cloned into vectors using the Vazyme ClonExpress II One Step Cloning Kit (C113). Transformation of the recombinant plasmids was subsequently carried out in *Escherichia coli* DH10B. Correct transformants were selected, and the plasmids were validated to confirm the successful construction of the *ATF1* knockout plasmid. Similarly, the construction of the *ATF1* overexpression plasmid was achieved by amplifying fragments using primer pairs X3g-1 & Pg-1, X3g-2 & Pg-2, and P-6005 & P-6005 (where “X3” refers to the third neutral site on chromosome X of *Saccharomyces cerevisiae*, a site known to contribute to expanding the metabolic capacity of the yeast [14]), followed by ligation, transformation, and validation to obtain the correct plasmid.

*S. cerevisiae* transformation was performed according to the protocol described by Cao [15]. The exogenous DNA fragments used for knockout were obtained using fusion PCR technology. The knockout cassette was obtained by fusing the upstream and downstream gene fragments of *ATF1*, which were amplified separately. The expression cassette for the *ATF1* gene overexpression is shown in Figure S1. After transforming *S. cerevisiae*, the transformants were selected on SD (HIS) plates. The successful knockout and overexpression of *ATF1* were verified by extracting genomic DNA from the colonies. The *ATF1* knockout strain was designated as SCdA, and the *ATF1* overexpression strain was designated as SCpA.

### 2.4. Strains and Culture Conditions

The selected *S. cerevisiae* strains included the parental strain SC, the gene-knockout strain SCdA, and the overexpressing strain SCpA, all stored at −80 °C. Before use, strains were first activated on YPD solid medium and then inoculated into YPD liquid medium, followed by a 24 h incubation on a shaker at 28 °C. Following centrifugation of the culture (10,000 rpm, 10 min, 4 °C), the harvested cells were rinsed, reconstituted in sterile saline, and standardized to 7 log CFU/mL.

### 2.5. Preparation of the Sour Meat Model System

The sour meat model system was formulated according to the study by Cano-García [16].

Extraction of myofibrillar proteins [17]: Pork was trimmed of fat and connective tissue before being minced. The minced meat was combined with a five-fold volume of extraction buffer (0.1 M Tris-HCl, 20 mM EDTA, pH 7.0), followed by thorough homogenization until no visible particles remained. After centrifugation at 10,000 rpm for 30 min at 4 °C, the supernatant was collected and discarded.

Preparation of the sour meat model system [16]: Each 100 g of the model system contains 35 g of myofibrillar protein, 25 g of pork fat, 5 g of glucose, and 35 g of 0.1 M phosphate-buffered solution (pH 7.5). The mixture is blended and homogenized to prepare a sour meat model system. The inoculated group is treated by inoculating the model system with *S. cerevisiae* at a concentration of 1 × 10^7^ CFU/g, followed by fermentation at 30 °C for 7 days. The experimental groups were designated as follows: F, the sour meat model system prior to fermentation; C, a control group without *S. cerevisiae* inoculation; SCdA, a group inoculated with the *ATF1*-knockout strain (SCdA); SC, a group inoculated with the wild-type *S. cerevisiae*; and SCpA, a group inoculated with the *ATF1*-overexpressing strain (SCpA).

### 2.6. The Determination of pH, TBARS, Protein Concentration, and TCA-Soluble Peptide Content

Following homogenization and centrifugation of a 2 g sample mixed with 20 mL of water, the supernatant was collected for pH measurement with a benchtop pH meter (FE28; Mettler Toledo, Greifensee, Switzerland). The determination of TBARS was conducted as described by Cheng [18]. Homogenization of a 2 g sample was performed in 20 mL of phosphate buffer, followed by centrifugation (12,000× *g*, 6 min). We collected the resulting supernatant and quantified the protein concentration via the Bradford method. As described by Wang [19], a 2 g aliquot of the sample was mixed with 8 mL of 10% TCA solution, blended for 1 min, and then subjected to centrifugation to collect the supernatant for measuring the content of TCA-soluble peptides. Using L-tyrosine as the standard, a standard curve was plotted. The content of TCA-soluble oligopeptides was expressed as μmol Tyr/g.

### 2.7. Determination of Fatty Acids

According to Pei’s method [20], total lipids were extracted from a 5 g sample. The sample was thoroughly combined with the extraction solution, concentrated at 35 °C by rotary evaporation, and the total lipid content was subsequently determined. Following derivatization of lipid fatty acids into fatty acid methyl esters with boron trifluoride-methanol, analysis was carried out on an Agilent 7890A GC-5975C MSD system (Santa Clara, CA, USA) equipped with an HP-5-MS capillary column (30 m × 0.25 mm × 0.25 μm). The specific conditions and parameters are detailed in the reference. The identification of fatty acids is combined with a comparative analysis of GC-MS retention times against those in a fatty acid standard by searching the NIST11 library. These compounds were then quantified based on their corresponding peak areas.

### 2.8. The Determination of Amino Acids and Electronic Tongue

A 3.0 g sample was blended with 12 mL of deionized water for homogenization before being treated with acetone. The mixture was allowed to stand for 10 min before centrifugation to collect the supernatant. Evaporated to dryness at 60 °C using a water bath, the supernatant was subsequently reconstituted in acetone and passed through a 0.22 μm membrane filter in preparation for subsequent analysis. Amino acid analysis was performed using the fully automatic amino acid analyzer LA8080 according to the manufacturer’s instructions. The standard used was an amino acid mixed standard (Sigma-Aldrich, St. Louis, MO, USA; Catalog number: AAS18). According to the formula TAV = C/T [21], the Taste Activity Value (TAV) is calculated to reflect the taste contribution of flavor amino acids in the sour meat model system. A TAV greater than 1 denotes a significant contribution to the overall taste. Here, C is the absolute concentration (mg/100g) of the compound, and T is its taste threshold (mg/100g).

The electronic tongue measurements were conducted according to the method of Liu [22]. The electronic tongue model TS-5000Z was purchased from Insent Inc. (Atsugi, Japan). Homogenization of a 30 g sample aliquot was performed with 100 mL of water. The resulting mixture was then passed through a 0.22 μm water-compatible filter membrane to prepare it for analysis.

### 2.9. Determination of Volatile Compounds

Analysis of volatile compounds in the samples was conducted through an SPME-GC-MS system. Full-scan (SCAN) mode was first employed to detect all flavor compounds in the sour meat, followed by selected ion monitoring (SIM) mode for precise quantification of esters. The procedure was performed as follows: We placed 2 g of the sample in a 20 mL headspace vial, added 20 µL of cyclohexanone internal standard (50 mg/L), and heated it at 60 °C for 30 min. We then collected volatiles using an SPME fiber for 40 min, which was subsequently desorbed for 10 min in the injector of an Agilent 7890A-5975C GC-MS system (Agilent Technologies, Inc.; Santa Clara, CA, USA) and analyzed on an HP-5MS capillary column (30 m × 250 µm × 0.25 µm). The specific conditions were the same as those described by Liu [23]. Substance identification was performed using the NIST 11 standard library for automatic retrieval. Calculation of the Kovats retention indices (RI) for all volatile compounds was performed using the retention time data of a C7–C30 n-alkane homolog mixture. Quantification was performed via comparison of individual peak areas to that of the internal standard, with results given in micrograms per 100 g (µg/100g).

### 2.10. Transcriptomics Sequencing

After fermentation at 30 °C for 7 d, aliquots (5 g) of each sample from the SCdA and SCpA groups were aseptically transferred into sterile 10 mL centrifuge tubes and cryopreserved at −80 °C. The samples were transported to Biomarker Technologies Corporation (Beijing, China) using dry ice for Illumina transcriptomics sequencing. The purity and concentration of RNA were assessed using a NanoDrop 2000 spectrophotometer (Thermo Fisher Scientific Inc; Wilmington, DE, USA), while the integrity of RNA was precisely evaluated using an Agilent 2100 Bioanalyzer/LabChip GX (Agilent Technologies, Inc.; Santa Clara, CA, USA). After sample qualification, libraries were constructed. Following library quality inspection, sequencing was performed on the Illumina NovaSeq 6000 platform in PE150 mode.

After obtaining the raw data, initial filtering was performed. Subsequently, the genome of *S. cerevisiae* S288C (GCF_000146045.2_R64) was used as a reference for alignment analysis using TopHat2. Following quality control of the transcriptomic data using RSeQC (v2.6.3) to analyze sequencing saturation, gene coverage, and duplicate reads, differentially expressed genes (DEGs) were identified. Gene expression levels, calculated as FPKM, were compared using the EdgeR package (v3.10) with screening thresholds set at |Fold Change| ≥ 1.5 and FDR < 0.05. The final DEGs were then functionally annotated and subjected to KEGG enrichment analysis.

### 2.11. Statistical Analysis

A completely randomized experimental design was employed in this study, and all experiments were independently repeated at least three times. Data were analyzed using SPSS version 27.0 (International Business Machines Corp., Armonk, NY, USA), and results are presented as means ± standard deviation (SD). Statistically significant differences among samples were determined by distinct alphabetic characters. Statistical significance was analyzed by one-way ANOVA followed by Duncan’s test at a significance level of *p* < 0.05. Figures and charts were created using Origin 2022 (OriginLab Corp., Northampton, MA, USA). The associated heatmaps were generated with TBtools-II v2.4 software.

## 3. Results and Discussion

### 3.1. Changes in pH and TBARS in the Sour Meat Model System

**Figure 1:**
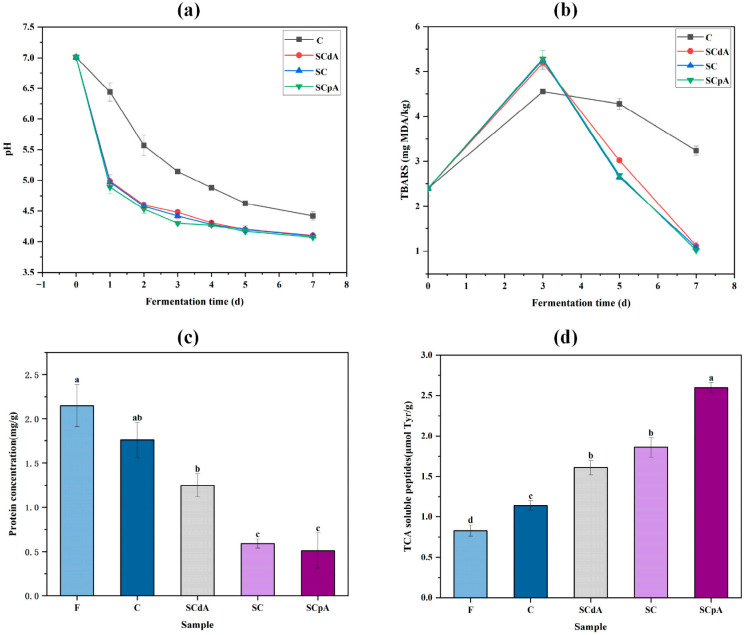
Changes of pH (**a**) and TBARS (**b**), content of protein (**c**) and TCA soluble peptide (**d**) in the sour meat model system. Note: F: the sour meat model system prior to fermentation; C: a control group without inoculation with SC; SCdA: a group inoculated with SCdA; SC: a group inoculated with untreated SC; SCpA: a group inoculated with SCpA. Different letters a–d indicated significant differences among different groups (*p* < 0.05).

Figure 1a demonstrated a marked decline in pH levels (*p* < 0.05) throughout the fermentation process. At the end of fermentation, the pH in every group had dropped to levels below 4.5. The drop in pH results from the bacterial metabolism of carbohydrates by lactic acid bacteria, which in turn drives lactic acid production [24]. A pH below 4.5 can suppress the proliferation of spoilage and pathogenic bacteria [25]. Therefore, the lower pH values of the four sample groups can delay food spoilage, facilitate preservation, and ensure food safety. Relative to the control (group C), a more pronounced reduction in pH was observed in the inoculated groups over the fermentation period (*p* < 0.05). The possible reasons are: on one hand, the inoculation of *S. cerevisiae* affects the growth of lactic acid bacteria by promoting their proliferation [24], while, on the other hand, the inoculation of *S. cerevisiae* makes carbohydrates more readily metabolized by lactic acid bacteria [26]. Therefore, by contributing to increased levels of organic acids, the inoculation of *S. cerevisiae* helps maintain a lower pH in the system.

TBARS is an indicator employed to assess the extent of lipid oxidation throughout fermentation [27]. Figure 1b shows that the TBARS level in the four fermentation groups initially increased and then decreased during fermentation. The TBARS values in the *S. cerevisiae*-inoculated samples showed a marked increase compared to the control at the mid-fermentation stage (*p* < 0.05), but subsequently declined to levels considerably below the control by the conclusion of the fermentation process (*p* < 0.05). Similar findings were also reported by Chaves-López [28]: in a myofibrillar protein sausage model inoculated with *S. cerevisiae*, protein consumption was significantly enhanced, leading to a more complex and intensified flavor profile. During the later stages of fermentation, *S. cerevisiae* was able to inhibit lipid oxidation to some extent, as evidenced by lower MDA content in *S. cerevisiae*-inoculated groups. This may be because *S. cerevisiae* promotes the production of MDA during the early stages of fermentation, which then interacts with other components such as nucleotides, nucleic acids, proteins, and other aldehydes to promote the accumulation of aromatic substances [29,30]. Therefore, *S. cerevisiae* promotes fat oxidation to enhance aroma in the beginning of the sour meat model fermentation, while it inhibits fat oxidation during the later stages to prevent the development of off-flavors.

### 3.2. Changes in Protein Concentration and TCA Content in the Sour Meat Model System

As shown in Figure 1c, the protein concentration significantly decreased (*p* < 0.05) in all groups after fermentation. The decrease in protein concentration is attributed to the breakdown of proteins into peptides, amino acids, aldehydes, and other low-molecular-weight components [31]. The protein concentration in *S. cerevisiae*-inoculated groups was significantly lower than that in control group C (*p* < 0.05). This may be due to the introduction of *S. cerevisiae* promoting protein degradation [30]. In summary, inoculation with *S. cerevisiae* facilitates the decomposition and transformation of proteins in the sour meat model system.

TCA-soluble peptides are important components contributing to the umami flavor of acid meat [32]. Figure 1d shows that the content of TCA-soluble oligopeptides significantly increased (*p* < 0.05) in all groups after fermentation. The TCA-soluble oligopeptide content in *S. cerevisiae*-inoculated groups was significantly higher than that in control group C, indicating that more myofibrillar proteins were degraded by endogenous proteases and microbial proteases in *S. cerevisiae*-inoculated groups. Jones’s research found that *S. cerevisiae* contains proteases and aminopeptidases, which can hydrolyze proteins to produce peptides and amino acids [33], aligning with our study results. The continuous accumulation of soluble peptides in *S. cerevisiae*-inoculated groups may enhance the potential for achieving desirable flavor in meat products.

### 3.3. Changes in Free Fatty Acid Content in the Sour Meat Model System

Lipases catalyze the hydrolysis of lipids in meat products, generating free fatty acids (FFA). [34]. The fatty acid content was found to be significantly elevated in all sour meat model systems following the fermentation process. Moreover, the *S. cerevisiae*-inoculated groups yielded a significantly greater fatty acid content than the control group C (*p* < 0.05) (Table S2). In general, within each group, palmitic acid, stearic acid, and oleic acid accounted for relatively high proportions. Additionally, the fatty acid compositional profile demonstrated a distinct pattern: polyunsaturated fatty acids (PUFA) showed the highest concentration, followed by saturated fatty acids (SFA), with monounsaturated fatty acids (MUFA) accounting for the lowest proportion. Consistent findings have been reported in other studies. For instance, in Olivares’ research [35], as the fermentation of sausages progressed, the content of FFA significantly increased (*p* < 0.01). Notably, sausages inoculated with *S*. *cerevisiae* exhibited the highest free fatty acid (FFA) content. Additionally, an observable trend emerged across treatment groups, indicating that unsaturated fatty acid levels exceeded those of saturated fatty acids. Palmitic acid and oleic acid were identified as the predominant fatty acids in all samples. During the fermentation process, unsaturated fatty acids undergo oxidative degradation, generating volatile compounds that influence the flavor profile of meat products. Gianelli [36] demonstrated that Mallorca Black Pork Sobrassada contains a significant amount of unsaturated fatty acids. As fermentation progresses, these FFAs are oxidized, resulting in a marked rise in the levels of volatile compounds within the product. In our sour meat model system, the SCpA group exhibited the highest content of unsaturated fatty acids, indicating that this group may produce higher levels of volatile compounds, which could potentially influence the flavor profile of the sour meat.

### 3.4. Changes in Free Amino Acid Content and Electronic Tongue Analysis in the Sour Meat Model System

**Figure 2:**
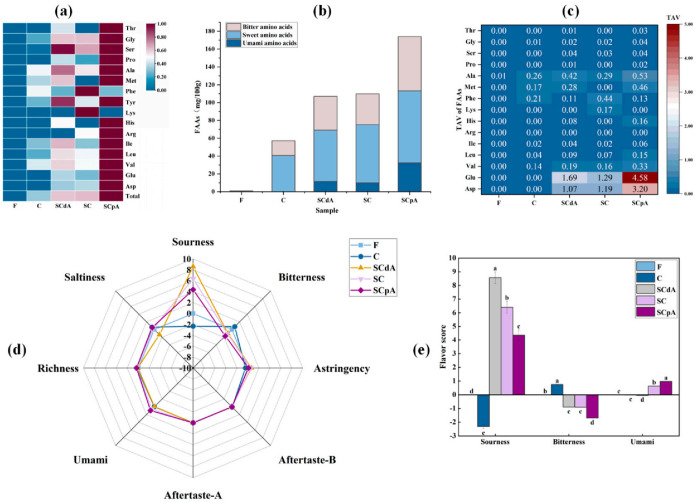
(**a**) Changes of FAAs content before and after fermentation in different groups; (**b**) Taste activity value (TAV) of FAAs; (**c**) Classification of taste-presenting FAAs; (**d**,**e**) Electronic tongue. Note: Total: Total free amino acid. Different letters a–d indicated significant differences among different groups (*p* < 0.05).

As shown in Figure 2a, relative to the fresh sample F, the total free amino acid content significantly increased (*p* < 0.05) in all groups after fermentation. Moreover, *S. cerevisiae*-inoculated groups demonstrated significantly elevated levels (*p* < 0.05) relative to control group C. Our results corroborate the findings of Nie [37], who attributed this phenomenon to the proteolytic or aminopeptidase activity of *S*. *cerevisiae* on meat proteins, leading to the breakdown of proteins and production of free amino acids. In *S. cerevisiae*-inoculated groups, the SCpA group exhibited the highest total free amino acid (TAA) content, followed by the SC group, and then the SCdA group. This phenomenon may be attributed to the overexpression of the *ATF1* gene, which leads to the upregulation of certain genes involved in the regulation of amino acid biosynthesis pathways, thereby promoting amino acid synthesis and indirectly resulting in significant accumulation of amino acids.

In the study by Chaves-López, the proteolytic activity of *S. cerevisiae* strains in the myofibrillar protein model led to the accumulation of amino acids such as Glu and Ala [28]. Similarly, as shown in Figure 2b, our findings demonstrate that the content of sweet amino acids increased post-fermentation compared to pre-fermentation levels. Notably, the concentration in the S. cerevisiae-inoculated groups was significantly higher than that in the control group (*p* < 0.05). After fermentation, compared to the control group, the *S. cerevisiae*-inoculated groups produced substantial amounts of Asp and Glu, two free amino acids that impart a savory (umami) taste. As shown in Figure 2c, both amino acids showed a taste activity value (TAV) > 1, indicating that Asp and Glu are the primary taste-active amino acids in this simulation system. Asp and Glu exhibited the highest concentrations in the SCpA group, and similarly, the content of amino acids such as Met and Val was also highest in the SCpA group (*p* < 0.05). The accumulation of these free amino acids will contribute to improving the flavor and nutritional value of the products during the sour meat fermentation process [38].

Electronic tongue analysis results for pre- and post-fermentation stages in the model system are shown in Figure 2d,e. As can be seen from the figure, after fermentation, the sourness of *S. cerevisiae*-inoculated groups was significantly higher than that of the control group C, while the bitterness was significantly lower compared to the control group. In *S. cerevisiae*-inoculated groups, the umami taste was highest in the SCpA group, while sourness and bitterness were the lowest (*p* < 0.05). Overall, the SCpA group exhibited the highest level of overall acceptability, which may be attributed to its rich composition of flavor substances.

### 3.5. Changes in Volatile Compounds in the Sour Meat Model System

**Figure 3:**
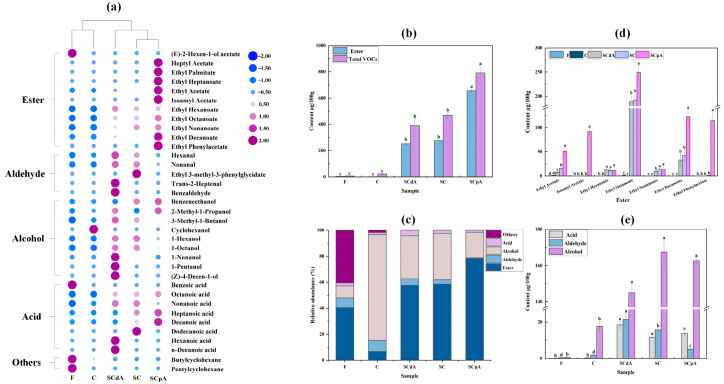
(**a**) Heat map of volatile compounds in the sour meat model system; (**b**) the contents of ester and total VOCs in different groups before and after fermentation; (**c**) the relative abundance of different classes of VOCs; (**d**) the contents of key esters in different groups before and after fermentation; (**e**) the contents of acid, aldehyde and alcohol in different groups before and after fermentation. Note: Data used in figures (**a**–**c**,**e**) were obtained using the SCAN mode; data in figure (**d**) were acquired using the SIM mode. Different letters a–d indicated significant differences among different groups (*p* < 0.05).

Figure 3a shows that a total of 35 volatile compounds were identified in the model system before and after fermentation, including 9 alcohols, 5 aldehydes, 8 acids, 11 esters, and 2 other compounds. These substances, which contribute significantly to the unique flavor profile of sour meat, are produced through lipid oxidation, proteolysis, and amino acid catabolism [39]. Prior to fermentation, 8 compounds were detected in sample F. After 7 days of fermentation, 10, 21, 20, and 21 compounds were identified in groups C, SCdA, SC, and SCpA, respectively. Compared with the pre-fermentation stage, both the variety and the quantity of compounds increased. According to Figure 3b, the total content of volatile substances in *S. cerevisiae*-inoculated group samples was significantly elevated compared to the control group C and the pre-fermentation sample F, with the SCpA group showing the most significant difference (*p* < 0.05). Additionally, the ester content in the SCpA group was also significantly higher compared to the other groups. Figure 3c shows that post-fermentation flavor compounds in the control group were predominantly alcohols, whereas esters were the dominant compounds in *S. cerevisiae*-inoculated groups. Notably, the SCpA group displayed the highest relative abundance of esters among all *S. cerevisiae*-inoculated groups. This indicates that inoculating with *S. cerevisiae* can alter the production of volatile substances in the model system, particularly having a significant effect on the esters.

The semi-quantitative results obtained in SCAN mode are susceptible to matrix interference. Therefore, we further performed precise quantification of key esters in sour meat under the selected ion monitoring (SIM) mode, particularly focusing on ethyl acetate and isoamyl acetate, which are produced by the AATases encoded by the *S. cerevisiae ATF1* gene. According to Figure 3d, esters in the *S. cerevisiae*-inoculated groups predominantly comprised ethyl decanoate, ethyl octanoate, and ethyl hexanoate. Additionally, in the SCpA group, significant amounts of ethyl phenylacetate, isoamyl acetate, and ethyl acetate were detected. These esters contribute a rich aroma profile (including fruity and floral notes) to the model system [40]. After fermentation, the ethyl acetate content among the groups was ranked as SCpA > SC > SCdA. Specifically, compared to the SC group, the ethyl acetate content in the SCdA group decreased by 61.23%, while in the SCpA group, it increased by 70.15%. Moreover, isoamyl acetate was detected only in the SCpA group (*p* < 0.05). This indicates that an increase in the expression level of the *S. cerevisiae ATF1* gene can enhance the content of ethyl acetate and isoamyl acetate in the sour meat model system. These two esters impart a fruity sweetness to the product. Previous studies have reported that overexpression of the *ATF1* gene in *S. cerevisiae* can increase the acetate ester content in alcoholic beverages [41]. Our experimental results also confirm a similar effect in fermented meat systems.

Alcohols are essential components of fermented meat flavor, primarily originating from carbohydrate metabolism, lipid oxidation, and amino acid catabolism [42]. According to Figure 3a,e, compared to the pre-fermentation stage, both the variety and the quantity of alcohols increased in each group post-fermentation. Specifically, the SCpA and SC groups exhibited the highest levels of alcohols, particularly benzeneethanol (*p* < 0.05). Benzeneethanol, as one of the alcohols that influence the flavor of fermented meat, is a volatile alcohol with a rose-like aroma [43]. However, in the SCdA group, the content of other alcohols was significantly higher than that in the SCpA and SC groups, particularly 3-methyl-1-butanol (*p* < 0.05). This may be due to the absence of the *S. cerevisiae ATF1* gene in the SCdA group, which prevents the production of isoamyl acetate in the simulated system, leading to a significant accumulation of 3-methyl-1-butanol. This is consistent with the findings of Li et al. in their study on liquor, which showed that *ATF1* deletion significantly decreases the content of ethyl esters while increasing the content of alcohols [44]. After fermentation, the content and variety of aldehydes and acids increased to different extents in each group, with significantly higher levels observed in *S. cerevisiae*-inoculated groups compared to group C (*p* < 0.05). This indicates that inoculating with *S. cerevisiae* can enhance the content of aldehydes and acids in the sour meat model system. In sour meat, the primary aldehydes are aliphatic aldehydes, which in meat products are typically the oxidation products of unsaturated fatty acids caused by endogenous enzymes within fat tissues or muscle tissues [43,45]. In the sour meat model system inoculated with *S. cerevisiae*, the aldehyde content across the groups was ranked as SCdA > SC > SCpA (*p* < 0.05). This is likely because the overexpression of *ATF1* in the SCpA group promotes the conversion of more fatty acids into fatty acyl-CoA, which in turn increases the production of fatty acid esters. This inhibits the autoxidation of fatty acids, leading to a reduction in aliphatic aldehyde content.

Overall, inoculation with *S. cerevisiae* significantly altered the production profile of volatile compounds in the model system, particularly enhancing both the concentration and diversity of esters. Notably, the *SCpA* group exhibited the most pronounced effect. The upregulation of the *ATF1* gene in *S. cerevisiae* significantly elevates the concentrations of ethyl acetate and isoamyl acetate in the simulated sour meat system, thereby imparting a distinct fruity-sweet aroma profile to the final product. Additionally, inoculation of *S. cerevisiae* enhances the concentrations of alcohols, aldehydes, and organic acids in the system.

### 3.6. Transcriptomic and Major Metabolic Pathway Analysis of the Sour Meat Model System

**Figure 4:**
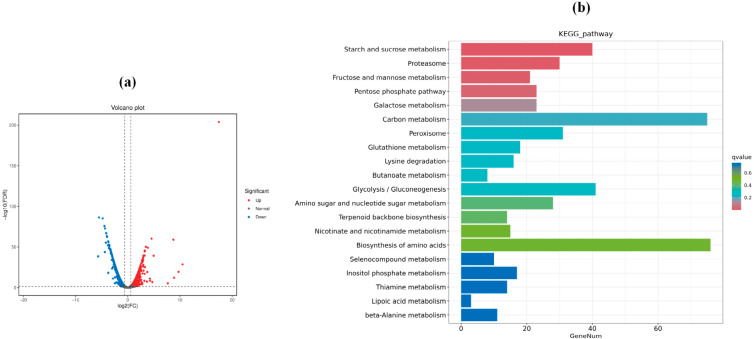
(**a**) Volcano plot of DEGs between the *ATF1*-overexpressing strain (SCpA) and *ATF1*-knockout strain (SCdA) in *S. cerevisiae*; (**b**) KEGG enrichment histogram of DEGs between SCdA and SCpA.

Transcriptomic analysis of *S. cerevisiae* fermentations in the SCdA and SCpA groups was performed, with a Fold Change ≥ 1.5 and FDR < 0.05 set as the criteria for identifying differentially expressed genes (DEGs). A total of 3197 DEGs were detected, as shown in Figure 4a, including 1639 upregulated genes (red) and 1558 downregulated genes (green). As shown in Figure 4b, the top 20 significantly enriched pathways were characterized based on KEGG enrichment analysis. The DEGs were mainly enriched in pathways such as carbon metabolism, biosynthesis of amino acids, glycolysis/gluconeogenesis, and starch and sucrose metabolism. The secondary products of these metabolic pathways, such as fatty acids, alcohols, esters, and amino acids, all contribute to the production of aroma compounds in *S. cerevisiae* [46].

**Figure 5:**
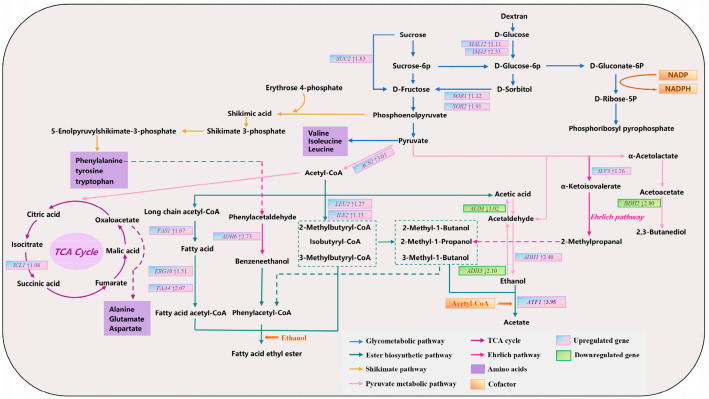
Fold change in DEGs in the aroma metabolism pathway between SCdA and SCpA groups. Note: The unit for differential gene expression fold change is log2 FC.

To systematically elucidate the formation mechanism of unique flavor compounds in the sour meat model system by the SCpA group, we conducted an in-depth analysis of key enriched metabolic pathways associated with DEGs, based on the detection results of volatile compounds (Figure 5). Analysis revealed that through systematic regulation of key pathways such as central carbon metabolism, the Ehrlich pathway, and ester synthesis, the SCpA group redirected the metabolic flux of flavor precursors, ultimately leading to the formation of its distinct aromatic profile.

The enhancement of central carbon metabolism provided a substantial material basis for the synthesis of flavor precursors. As shown in Figure 5, in the sugar metabolic pathways, the sucrase gene *SUC2*, as well as genes involved in glucose and fructose utilization (including *MAL12*, *IMA3*, *SOR1*, and *SOR2*), were significantly upregulated in the SCpA group. The synergistic action of these genes markedly improved the strain’s efficiency in carbon source utilization, thereby supplying more abundant phosphoenolpyruvate (PEP) and pyruvate for downstream metabolism. These two key metabolic nodes serve as a “crossroads” for flavor compound synthesis: on one hand, PEP provides precursors for the synthesis of aromatic amino acids such as phenylalanine via the shikimate pathway; on the other hand, pyruvate not only serves as the starting point for the synthesis of branched-chain amino acids like valine and leucine, but also acts as the entry point to TCA cycle. It is noteworthy that the upregulation of *ICL1*, a key gene in the TCA cycle, in the SCpA group promoted the accumulation of oxaloacetate, thereby ensuring adequate substrate availability for the synthesis of amino acids such as aspartate and glutamate [47]. This synergistic enhancement of upstream metabolic pathways established an abundant precursor pool for the subsequent synthesis of flavor compounds, including higher alcohols and esters, which is consistent with the significantly higher total free amino acid (FAA) content observed in the SCpA group compared to other groups.

Differential regulation of the Ehrlich and pyruvate metabolic pathways precisely shaped the composition of higher alcohols. Regarding alcohol biosynthesis, alcohol dehydrogenases encoded by *ADH* genes catalyze the conversion between aldehydes and alcohols. Increased expression of genes such as *ADH6* has been reported to be associated with elevated benzeneethanol production, while enhanced expression of genes including *ADH3* is linked to increased synthesis of higher alcohols such as 3-methyl-1-butanol and 2-methyl-1-propanol [48]. Overexpression of the *ILV3* gene facilitates the conversion of pyruvate to α-ketoisovalerate, which is subsequently decarboxylated to 2-methylpropanal and ultimately reduced to 2-methyl-1-propanol [49]. In the SCpA group, the Ehrlich pathway and pyruvate metabolic pathway exhibited a highly specific regulatory pattern. On one hand, both *ILV3*, which is closely associated with 2-methyl-1-propanol synthesis, and the critical gene *ADH6* responsible for benzeneethanol production were significantly upregulated. This finding is closely aligned with the experimental results showing the highest detected levels of benzeneethanol and 2-methyl-1-propanol in the SCpA group. On the other hand, the alcohol dehydrogenase gene *ADH3*, which catalyzes the formation of various higher alcohols such as 3-methyl-1-butanol, was markedly downregulated. This differential expression pattern, characterized by both increases and decreases among members of the alcohol dehydrogenase gene family, precisely modulated the conversion efficiency of different precursors into their corresponding higher alcohols, thereby reprogramming the composition profile of higher alcohols.

The acetyl-CoA-centered ester biosynthesis network was significantly activated. Esters represent important flavor contributors in the system, and their synthesis efficiency depends on the supply of two major precursor types: alcohols and acyl-CoAs. In the SCpA group, both of these supply lines were enhanced. First, regarding the supply of acyl-CoAs, the upregulation of *ACS2* directly enhanced the synthesis capacity of acetyl-CoA [50]. Concurrently, upregulated expression of *FAS1*, *ERG10*, and *FAA4* promoted the generation of long-chain fatty acids and their activated products—long-chain acyl-CoAs. Meanwhile, increased expression of *LEU2* and *ILE2* boosted the production of precursors such as 2-methylbutyryl-CoA. The downregulation of *BDH2* reduced the conversion of acetoacetate to 2,3-butanediol, leading to the accumulation of acetoacetate [51]. We speculate that this may result in more pyruvate being converted into acetyl-CoA, thereby increasing the supply of acetyl-CoA available for ester synthesis. Furthermore, regarding alcohol supply, the upregulation of *ADH1* ensured sufficient ethanol substrate. Ultimately, in the esterification step, the expression of *ATF1*—a key gene encoding alcohol acetyltransferase—was significantly upregulated. This enzyme efficiently utilizes the aforementioned acetyl-CoA and alcohols to catalyze the extensive synthesis of acetate esters such as ethyl acetate and isoamyl acetate [52]. This result is also consistent with the previously reported inverse correlation between *ALD3* expression and ethyl acetate content [53]. In summary, through the coordinated enhancement of diversified acyl-CoA supply, generation of key alcohol substrates, and the final catalytic step of esterification, the SCpA group significantly elevated the overall synthesis capacity of ester flavor compounds via the synergistic effects of multiple genes. This finding is closely aligned with the flavor detection results.

## 4. Conclusions

Through a sour meat model system, this research demonstrates that modifying the *ATF1* gene in *Saccharomyces cerevisiae* substantially affects acetate ester synthesis. Overexpression of *ATF1* increased the strain’s esterification ability, raising levels of ethyl acetate and isoamyl acetate, while its knockout notably reduced ethyl acetate content. Transcriptomics further showed that *ATF1* overexpression not only enhanced esterification directly but also upregulated key genes in central carbon metabolism, amino acid synthesis, and precursor supply (e.g., *SUC2*, *ICL1*, *ACS2*, *ADH1*). This indicates that changes in *ATF1* expression disturb a wider metabolic network, working together to boost alcohol and acyl-CoA precursor accumulation and ultimately increase acetate ester yield. These findings position *ATF1* as a useful genetic target for steering acetate ester flavor in the sour meat model system. However, because the model differs from actual sour meat fermentation in matrix structure, microbial interactions, and environmental factors, industrial applicability will require further testing at pilot and production scales.

## Figures and Tables

**Table 1:** Strains and plasmids.

Strains and Plasmids		Genotype or Sequence	Source
**Strains**	*Saccharomyces cerevisiae* SC	*MATa; MAL2-8c; SUC2; his 3△1; ura3-52; gal80△;XI-5::*P*_TEF1_*-*Cas9*-T*_CYC1_*	Yongjin Zhou’s lab; Dalian Institute of Chemical Physics, CAS
	*S. cerevisiae* SCdA	*MATa; MAL2-8c; SUC2; his3△1; ura3-52; gal80△; XI-5::*P*_TEF1_*-*Cas9*-T*_CYC1_*; △*atf1*	This research
	*S.cerevisiae* SCpA	*MATa; MAL2-8c; SUC2; his3△1; ura3-52; gal80△; XI-5::*P*_TEF1_-Cas9*-T*_CYC1_*; X3*::*P*_TPI1_-ATF1*-T*_CYC1_*	This research
	*Escherichia* *coli* DH5a	*F-, φ80d/lacZ* *△M15,* *△(lacZYA-argF) U169, deoR, recAl, endAl, hsdR17(rk, mk^+^), phoA, supE44, λ^-^, thi-1, gyrA96, relA1*	Takara
**Plasmid**	pgRNA-X3	*URA3, 2 µm, Amp^R^*, gRNA1-X3, gRNA2-X3	This research
	pgRNA-ATF1	*URA3, 2 µm, Amp^R^*, gRNA1-ATF1 gRNA2-ATF1	This research

## Data Availability

The original contributions presented in the study are included in the article/Supplementary Material, further inquiries can be directed to the corresponding author.

## Supplementary Materials

The following supporting information can be downloaded at: https://www.mdpi.com/article/10.3390/foods15020378/s1, Figure S1: Construction of *ATF1* gene deletion and overexpression strains. Table S1: Oligonucleotide primers. Table S2: Free fatty acid content in model system of sour meat.
