# Supplementary material for: Genetic Modulation of ATF1 in Saccharomyces cerevisiae for Enhanced Acetate Ester Production and Flavor Profile in a Sour Meat Model System
Source: Foods. 2026 Jan 21;15(2):378. doi: 10.3390/foods15020378 (PMC12841429; doi:10.3390/foods15020378)
Supplement: Supplementary file 1 [file foods-15-00378-s001.zip › foods-4070364-supplementary.pdf]

Table S1

Table S1 Oligonucleotide primers

Number	Oligonucleotide primers	Sequence(5′-3′)	Function
(1)	P-6005	GATCATTTATCTTTCACTGCGGAGAAG	Amplification Vector 6005
(2)	Ag-1	AAACTTCTCCGCAGTGAAAGATAAATGATCCAAATATTCTATATGCGATTGTTTTAGAGCTAGAAATAG	Amplification with primers (2) and (3) yielded the Ag1 fragment;
(3)	Pg-1	GCGGTTAGCTCCTTCGGTCCTCCGATCGTTGTCAGAAGTAAGTTGGCCGCAGTGTTATC	Amplification with primers (4) and (3) yielded the X3g1 fragment.
(4)	X3g-1	AAACTTCTCCGCAGTGAAAGATAAATGATCATCGATTGCTCCACTCATAAGGTTTTAGAGCTAGAAATAG	
(5)	Ag-2	AAACTTCTCCGCAGTGAAAGATAAATGATCCTCCGTTTTTACATGTTTGTGTTTTAGAGCTAGAAATAG	Amplification with primers (5) and (6) yielded the Ag2 fragment;
(6)	Pg-2	GATAACACTGCGGCCAACTTACTTCTGACAACGATCGGAGGACCGAAGGAGCTAACCGC	Amplification with primers (7) and (6) yielded the X3g2 fragment.
(7)	X3g-2	AAACTTCTCCGCAGTGAAAGATAAATGATCCAAGAACAAGCTTCAACTTTGTTTTAGAGCTAGAAATAG	
(8)	gRNA-A-YZ-F	CGAGATCTTTGTGTTCGGTTACCC	Verification of the ATF1 knockout plasmid.
(9)	gRNA-A-YZ-R	CTCAATGAACGAGCTCGAGCG	
(10)	gRNA-X3-YZ-F	CGAGATCTTTGTGTTCGGTTACCC	Verification of the ATF1 overexpression plasmid.
(11)	gRNA-X3-YZ-R	CTCAATGAACGAGCTCGAGCG	
(12)	ATF1-up-R	CGAATAATATCAGTCAAGCATCATGTGAGATGAGAGCTGATAAATTGATGGTATTTGTG	Amplification with primers (12) and (13) yielded ATF1-up;
(13)	ATF1-up-F	CCGAAGAAATGCAAAGAAGTAAGGTTAG	
(14)	ATF1-down-R	TTAAAAATATCTCTCCTTGCTTAAGG	Amplification with primers (14) and (15) yielded ATF1-down;
(15)	ATF1-down-F	CACAAATACCATCAATTTATCAGCTCTCATCTCACATGATGCTTGACTGATATTATTCG	Amplification with primers (13) and (14) yielded the ATF1-up+ATF1-down fragment.
(16)	ATF1-R	GTAAGCGTGACATAACTAATTACATGACTAAGGGCCTAAAAGGAGAGCTTTGTAAATG	Amplification of the ATF1 fragment.
(17)	ATF1-F	CTACAAAAAACACATACATAAACTAAAAATGAATGAAATCGATGAGAAAAATCAGGCCC	
(18)	X3-up-F	CCATGTACTCAAGCTCGTCTCC	Amplification with primers (18) and (19) yielded the X3up fragment;
(19)	X3-up-R	GTGCCTCCCTTGACAAAGGACTGAATCGTTGTGCTGGAGTAGTTGG	
(20)	X3-down-F	CCAACTACTCCAGCACAACGATTCAGTCCT	Amplification with primers

		TTGTCAAGGGAGGCAC	(20) and (21) yielded the X3down fragment;
(21)	X3-down-R	GCCTGTTGCTGCTCTTGAATGG	
(22)	X3-TPIp -F	CCAACTACTCCAGCACAACGATTCAGTTTAAAGATTACGGATATTTAAC	Amplification with primers (22) and (23) yielded the TPIp fragment;
(23)	TPIp-R-A	GGGCCTGATTTTTCTCATCGATTTCATTCATTTTTAGTTTATGTATGTGTTTTTTGTAG	Amplification with primers (24) and (25) yielded the CYC1t fragment;
(24)	A-CYC1t-F	CATTTACAAAGCTCTCCTTTTAGGCCCTTAGTCATGTAATTAGTTATGTCACGCTTAC	
(25)	CYC1t-R-X3	GTGCCTCCCTTGACAAAGGACGCAAATTAAAGCCTTCGAGCGTCC	Amplification with primers (18) and (23) yielded the TPIp+X3up fragment; Amplification with primers (24) and (21) yielded the CYC1t+X3down fragment; Amplification with primers (22) and (25) yielded the TPIp+ATF1+CYC1t fragment.
(26)	YZ-ATF1-F	GTGCCTCCCTTGACAAAGGACTGAATCGTTGTGCTGGAGTAGTTGG	Verification of SCdA transformants.
(27)	YZ- ATF1-R	CCAACTACTCCAGCACAACGATTCAGTCCTTTGTCAAGGGAGGCAC	
(28)	YZ-X3-F	CTGGTGTTGCCTCTAACATATACC	Verification of SCpA transformants.
(29)	YZ- X3-R	GTGCATACGCTTACGCACACACAC	

Figure S1

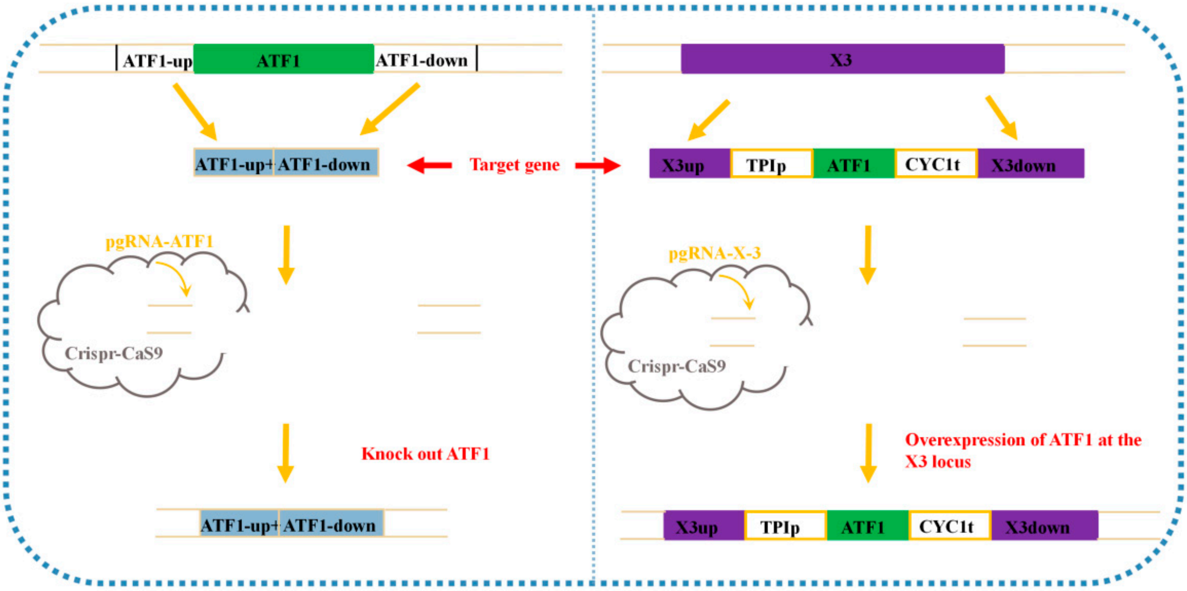

Fig S1 Construction of *ATF1* gene deletion and overexpression strains.

Table S2

Table S2 Free fatty acid content in model system of sour meat

Free fatty acid	Samples				
(g/100g)	F	C	SCdA	SC	SCpA
C14:0	1.09±0.01^{d}	1.22±0.10^{d}	1.50±0.05^{c}	1.82±0.06^{d}	1.75±0.03^{b}
C16:0	11.56±1.31^{c}	15.40±0.39^{b}	17.98±0.99^{b}	22.04±0.94^{a}	21.64±0.35^{a}
C18:0	11.06±0.69^{c}	14.61±0.35^{b}	15.29±0.79^{ab}	15.45±1.19^{ab}	18.43±1.36^{a}
SFA	23.71±1.98^{d}	31.23±0.85^{c}	34.77±0.25^{bc}	39.31±2.07^{ab}	41.83±0.98^{a}
C16:1	1.41±0.16^{b}	1.77±0.21^{ab}	1.97±0.07^{ab}	2.57±0.43^{a}	2.51±0.44^{ab}
C20:1	0.36±0.03^{b}	0.92±0.08^{a}	0.94±0.01^{a}	0.80±0.15^{a}	0.93±0.05^{a}
MUFA	1.78±0.19^{b}	2.69±0.13^{a}	2.88±0.06^{a}	3.37±0.27^{a}	3.44±0.39^{a}
C20:2	0.34±0.01^{b}	0.00±0.00^{c}	0.81±0.08^{a}	0.00±0.00	0.00±0.00^{c}
C18:1 n-9	20.04±0.38^{c}	27.67±1.51^{b}	33.46±0.52^{a}	33.89±1.66^{a}	36.27±1.01^{a}
C18:2 n-6	9.97±0.28^{d}	11.68±0.38^{c}	14.46±0.54^{b}	15.95±0.07^{ab}	16.28±0.71^{a}
C18:3 n-3	0.00±0.00^{c}	0.00±0.00^{c}	0.27±0.04^{b}	0.46±0.04^{a}	0.36±0.03^{ab}
PUFA	30.36±0.09^{c}	39.35±1.89^{b}	49.02±1.10^{a}	50.31±1.55^{a}	52.93±0.26^{a}
Total	55.85±1.02^{d}	73.27±2.13^{c}	86.67±2.19^{b}	92.99±3.02^{a}	98.20±2.22^{a}

Note: Different letters a-d indicated significant differences among different groups ($p < 0.05$).

SFA: Saturated fatty acid; MUFA: Monounsaturated fatty acids; PUFA: Polyunsaturated fatty acids; Total: Total fatty acid.
